# Links between energy budgets, somatic condition, and life history reveal heterogeneous energy management tactics in a group-living mesocarnivore

**DOI:** 10.1186/s40462-024-00453-1

**Published:** 2024-03-27

**Authors:** Julius G. Bright Ross, Andrew Markham, Christina D. Buesching, Catherine Hambly, John R. Speakman, David W. Macdonald, Chris Newman

**Affiliations:** 1 Wildlife Conservation Research Unit, Department of Biology, The Recanati-Kaplan Centre, University of Oxford, Tubney House, Abingdon Rd, Tubney, OX13 5QL UK; 2 Department of Computer Science, University of Oxford, Wolfson Building, Parks Road, Oxford, OX1 3QD UK; 3 The Irving K. Barber School of Arts and Sciences, The University of British Columbia, Okanagan Campus, Kelowna, BC Canada; 4 Institute of Biological and Environmental Sciences, University of Aberdeen, Tillydrone Avenue, Aberdeen, AB24 2TZ UK; 5 Centre for Energy Metabolism and Reproduction, Shenzhen Institutes of Advanced Technology, Chinese Academy of Sciences, Shenzhen, China

**Keywords:** Daily energy expenditure, Doubly-labelled water, Energy budgeting, Energetic ecology, Life-history trade-offs, Overall dynamic body acceleration

## Abstract

**Background:**

Optimal management of voluntary energy expenditure is crucial to the survival and reproductive success of wild animals. Nevertheless, a growing appreciation of inter-individual variation in the internal state driving movement suggests that individuals may follow different, yet equally optimal tactics under the same environmental conditions. However, few studies in wild populations have investigated the occurrence and demographic context of different contemporaneous energetic expenditure tactics. Here, we explore this neglected aspect of energy budgeting in order to determine the effect of life-history traits such as age and reproductive status on the co-occurrence of different energy-budgeting tactics in wild populations.

**Methods:**

We investigated inter-individual heterogeneity in energy expenditure within a wild population of European badgers (*Meles meles*) by quantifying individual overall dynamic body acceleration (ODBA, from tri-axial accelerometry collars) and total daily energy expenditure (DEE, from doubly-labelled water) during 6–9 day deployments and dosing periods over six different seasons (spring, summer, and autumn) in 2018–2019. We obtained ODBA values for 41 deployments (24 unique badgers) and DEE measurements for 41 dosings (22 unique badgers). We then evaluated correlations between these energetic metrics and computed individual ratios of ODBA/DEE as a proxy for the proportion of total energy spent on activity. We measured the impact of alternative ODBA/DEE ratios on body condition, and use survival models constructed using 29 years of demographic data from the same population to situate body-condition changes in the context of age and reproductive status.

**Results:**

Both ODBA and DEE were highly variable between individuals and exhibited season-specific relationships with individual body condition and life-history factors. DEE scaled allometrically with body weight, but only in summer and autumn; post-reproductive female badgers were lighter than other badgers during the spring but expended on average 350 kJ/day more than predicted from allometric scaling. Older badgers expended significantly less energy on movement during the summer than did younger adults. The ratio of ODBA to DEE (OD) provides a measure of proportional investment into movement. This ratio correlated more significantly with next-season body condition than either energetic metric did independently. However, the majority of individuals with high OD ratios were either younger badgers or reproductive females, for which lower body condition typically presented less of a mortality risk in previous analyses of this population.

**Conclusions:**

Within a single population under the same environmental conditions, we found wide inter-individual variation in both mechanical and total energy expenditure. The adoption of different tactics aligns with relationships between life-history parameters and mortality risk previously studied within the population. Crucially, younger badgers and reproductive females appeared able to tolerate energy expenditure tactics that depleted their body condition more than other badgers. These findings provide a mechanism by which differences in individual energetic context set by life history can maintain heterogeneity in wild populations, providing a wide range of potential energetic tactics under changing environmental conditions.

## Background

Energy is a limited currency in wild animal populations [1] and maintaining an appropriate balance between intake and expenditure is key to survival [2, 3]. Deviations from typical environmental conditions stress the equilibrium between an individual’s energy uptake and output [4], requiring plastic responses ranging through internal homeostasis [5], in-situ behavioural change [6], to migration [7]. The most fundamental form of plastic response for motile animals is movement, which provides an essential link between an animal’s internal state and its environment [8]. Many forms of human-induced rapid environmental change (HIREC) cause substantial energetic stress for wild populations [9]. This is a particular issue when climate change exacerbates thermoregulatory stresses and/or alters the availability of food supply, in terms of quantity, foraging distances, or greater temporal variation [10]. Moreover, obligate energy requirements vary between individuals according to life-history traits [11, 12], somatic condition [2], and allometric scaling [13], affecting each individual’s capacity to adapt to changing conditions. Despite the recognised importance of plastic behavioural responses such as movement modulation for ensuing population resilience under these changing conditions [14, 15], especially for non-vagile species or those constrained by other anthropogenic barriers [16], few studies have directly investigated how energy budgets vary between conspecifics experiencing the same environment simultaneously, instead assuming steady-state energy expenditure across populations (e.g., [17]).

All individuals within a population share the goals of survival and of maximising their lifetime reproductive success; nevertheless, even when experiencing identical contemporaneous weather conditions, predation risk, food availability, and social conditions, individuals may exhibit different energy budgets and activity regimens [18, 19]. Differences in basal metabolic rates [20, 21], energy needs relating to reproduction [22, 23], and age [24] are just some of the many factors that constrain energy expenditure in ways that are unique to each individual. This inter-individual variation in internal state within a single population mechanistically links individual movement decisions (when, why, and how much to move) to fitness, especially when the external environment experienced by a population is relatively homogeneous. After all, any variation in activity – whether different foraging tactics [18], social interactions [25], or even exploration of the environment [26] – is constrained by the reality that body condition reflects an individual’s remaining stores of energy, and when those stores run out, there is a greater risk that individual will die. Of particular interest within the range of energy-allocation tactics [27] is how individuals balance energy commitments to obligate homeostatic functions and the facultative expenditure of surplus energy on movement, giving rise to differential capacity to “pivot” energetically [18].

Studying fine-scale inter-individual energetic heterogeneity in the wild, where indirect calorimetry cannot be applied, has historically been difficult [28, 29]. However, over the past two decades, advances in bio-logging have made the Lagrangian approach to studying populations as a composite of individual movement pathways much more feasible [8, 30, 31]. Tri-axial accelerometry, in particular, can provide a direct measure of mechanical energy expenditure through movement (“overall dynamic body acceleration”, ODBA), even for animals with relatively small home ranges and/or habitat preferences that remain challenging for many GPS units [32, 33]. To study the expenditure of energy on movement in the context of the whole energy budget, ODBA can be paired with whole-organism energy expenditure (“daily energy expenditure”, DEE), which can be reliably quantified using doubly-labelled water [34]. In this study, we combine these two methods to measure inter-individual differences in total energy expenditure and allocation to movement, using the European badger (*Meles meles*) as a model species. We make use of 27 years of demographic data from the same population to situate individual energy budgeting within established demographic and body-condition predictors of survival to the next year.

In Northern Europe, badgers live in mixed-sex and -age groups within underground dens termed “setts”, emerging to forage at night. Badgers in high-density populations typically reproduce polygynandrously and promiscuously during a primary mating season in early spring (late February to early April, [35]), but delay implantation of blastocysts until late December [36]. Births occur in February and weaning in May. Badgers exhibit substantial changes in weight (and thus, body condition) throughout the year [4]. British badgers are lightest in summer when their primary food source, earthworms (*Lumbricus terrestris*) are scarce [37], and heaviest in autumn, when wetter conditions enhance earthworm availability and fruits and nuts become available as secondary food items [38], allowing the storage of substantial fat reserves to maintain physiological torpor throughout the winter months [39]. Within the same population—with the studied population comprising several dozen setts and approximately 240 individuals during the time of this study [40]—badgers concurrently exhibit substantial variation in inter-individual life-history [40], hormone profiles [41, 42], activity regimes [25, 43], and body condition profiles [4], even while experiencing rather homogeneous environmental conditions [44]. A previous analysis of the studied population found that low relative body condition indices (BCIs) have a strong predictive effect on the likelihood of a badger’s survival to the next year, but that the predictive power of BCI varies depending on key demographic factors [4]. For older badgers (approximately age 7 and above, where few badgers live beyond age 10; [40]), this predictive relationship was stronger, while for female badgers that had reproduced in a given year, BCI in spring and summer has no significant predictive ability on the individual’s likelihood of surviving to the next year. These poignant demographic markers of energetic frailty, therefore, provide a uniquely developed scaffold around the analysis of energy-budgeting trade-offs.

Here, we first examined (seasonally) which individual traits (sex, age, and body weight – chosen over body condition because of its allometric influence on energy expenditure) affect the active component of energy expenditure (ODBA) and whole energy budgets (DEE). If all individuals partition voluntary and involuntary expenditures similarly, irrespective of their different life-history contexts, both metrics should respond to the same drivers. Conversely, if individuals budget energy differently after accounting for individual traits, it would indicate a diversity of co-occurring energetic tactics, and/or demands, under the same prevailing conditions. We used the “fraction of energy spent on activity” (ODBA/DEE, “OD”) to characterise the variation in these energetic tactics. We also examined how these metrics affect subsequent body condition—and thus how activity rates translate into mortality risk through depleted body condition. To do so, we used models constructed with longitudinal data from this population—providing a view into the mosaic of individual energetic contexts making up populations in the wild.

## Methods

### Study site and animal captures

All data were collected in Wytham Woods (Oxfordshire, England; 51°46’N, 1°20’W), in the context of a 32-year demographic badger study [4, 45]. The study area comprises a 424-ha mixed-species woodland surrounded by the Thames River on three sides and by the A420 motorway on the fourth. No relevant predators are present in the study area, and while some degree of environmental heterogeneity exists between setts (e.g., ectoparasite loads, [46], or thermal properties, [47]), this study targeted badgers from a small cluster of three central setts in order to reduce the variation in environmental conditions experienced by the different individuals studied. This targeting allowed a *ceteris paribus* approach to quantifying movement and energy expenditure, where high variability in the measured indicators implied genuine differences between individual responses to near-identical extrinsic conditions. Captures were carried out during May (“spring”), September (“summer”), and November (“autumn”) of 2018–2019, following the same methodology as the other 30 years of the demographic study. We used string-trigger traps to capture badgers at three setts, then transported them in holding cages between 7:00 and 9:00 am to a central field station. Individuals were sedated with 0.2 mL ketamine hydrochloride/kg body weight by intramuscular injection [41, 48] and biometric data were recorded, including unique tattoo identity, weight (W, to 0.1 kg), body length (BL, to 5 mm), molar tooth wear (scale 1–5), and whether female badgers exhibited evidence of recent lactation. We computed a body condition index (BCI) as log_e_(W)/log_e_(BL). Individual age was tracked over the course of the demographic study using individual tattoos, and inferred at first capture (if not a cub) using molar tooth wear scores [40].

### Doubly-labelled water administration and collaring

We quantified daily energy expenditure (DEE, kJ/day) using the doubly-labelled water (DLW) technique [49]. This method has been validated by comparison to indirect calorimetry in a range of animals (e.g., [50]). To avoid the need to sedate study animals twice, we estimated individual body weight prior to injection while in holding cages (deducting the weight of the cage and handler), and then administered DLW (650,765 ppm ^18^O, 342,395 ppm ^2^H) on 65 occasions across 29 individual badgers (including repeat captures; maximum one administration per season, per year) via intramuscular injection according to the equation Mass_DLW (g)_ = 0.65*W (kg)*DIE/IE, where DIE is the desirable initial enrichment (in ppm) and IE is the injectate enrichment (both in ppm, [34]). We weighed syringes before and after injection (± 0.0001 g) to calculate the precise mass of DLW injected. After 3–3.5 h, we sedated these badgers and collected blood samples by jugular venipuncture into vacutainers with EDTA anticoagulant, then heat-sealed sub-samples into 100 µL glass capillaries, stored at room temperature. As we did not sedate animals prior to DLW injection, we could not collect background samples to deduce individual background isotope ratios, but instead used an average background enrichment from samples collected from 5 to 9 other individuals captured at the same social groups during that same 3-day trapping session. We released badgers at the sett where they had been captured, then attempted to recapture these same individuals 6–10 days later, chosen as an estimated time point before isotope elimination would reach background levels. Upon each release, every DLW-dosed badger was equipped with a tri-axial accelerometer attached to a padded dog collar. Tri-axial accelerometers were custom-made using SensorTile turn key sensor modules (STEVAL STLK01V1); code was written in C and compiled using ARM Keil MDK 5, then flashed to devices using a STLINKv2 programmer. Accelerometry data were sampled at 25 Hz, with no remote data acquisition in order to minimise battery drain. Upon recapturing these target badgers, we sedated them, identified them by tattoo, retrieved collars, and collected a second blood sample, which was sealed and stored as specified above. Time of isotope administration and blood sampling was recorded carefully.

### DEE estimation

Analysis of isotopic enrichment of blood samples was performed blind, using a Liquid Isotope Water Analyser (Los Gatos Research, USA) [51]. We vacuum-distilled blood from storage capillaries [52], then ran the resulting distillate alongside five lab standards for each isotope and 3 international standards to account for day-to day machine variation, then corrected delta values to ppm. We estimated a single DEE value for each individual per dosing (DEE_i_,where *i* represents an individual, from *i* = 1 to *i* = 41; this metric used in all subsequent analyses) using a single-pool model, as recommended for animals below 10 kg and used elsewhere for badgers [53]. Specifically, we used Eq. 7.17 from Speakman et al. [34].

### ODBA estimation

After downloading data from recovered collar SD cards, we computed a two-second basis ODBA (ODBA_2s_, where *2s* represents a 2 s window from *2s* = 1 to *2s* = 388,800 for the longest-deployed collar, from *2s* = 1 to *2s* = 259,200 for the shortest) time series (in *g*, ms^−2^) for each individual badger (*i*, from *i* = 1 to *i* = 41) as the L1-norm of the three channels of acceleration [33]:

1 $${{\text{ODBA}}}_{2{\text{s}},{\text{i}}}= |{\text{A}}_{\text{x,i}}| + |{\text{A}}_{\text{y,i}}| + |{\text{A}}_{\text{z,i}}|$$

where A_{x,y,z},i_ represent, for each badger, the difference between the mean and the midpoint of each channel over a two-second window, divided by 2^14^, the basis for *g* programmed for these accelerometers. We averaged these ODBA_2s,i_ values over each day of collar data for each individual to produce daily ODBA (ODBA_d__,__i_, where *d* represents each day, from *d* = 1 to *d* = 9 for the longest-deployed collar; from *d* = 1 to *d* = 6 for the shortest; n = 271 daily values across all individuals), and then averaged these in turn to produce a single average daily ODBA value for each individual badger (ODBA_i_ = avg(ODBA_d,i_), where *i* represents each individual, from *i* = 1 to *i* = 41; ODBA_i_ is the ODBA value used in all subsequent analyses) in order to permit comparison to DEE_i_. We omitted first and last days of deployment from each ODBA_i_ calculation, due to a significant reduction in first-day post-release activity and given that the badger spends at least a portion of the last day of deployment in a cage.

### Drivers of ODBA and DEE variation

We used linear regressions in R (Version 3.5.3, [54]) to test intrinsic drivers of energetic metrics. First, we considered whether either metric exhibited allometric scaling, permitting the scaling relationship to change by season (where *β*_*s*_ is season-dependent):

2 $${Metric}_{i} ({DEE}_{i}\; or\; {ODBA}_{i}) = {\alpha }_{1}{{W}_{i}}^{{\beta }_{s}}$$

To suit a linear modelling framework, we log-transformed the response and predictor variables:

3 $${\text{ln}}({metric}_{i})={{\text{ln}}(\alpha }_{1})+{\beta }_{s}\cdot{\text{ln}}({W}_{i})$$

**Fig. 1:**
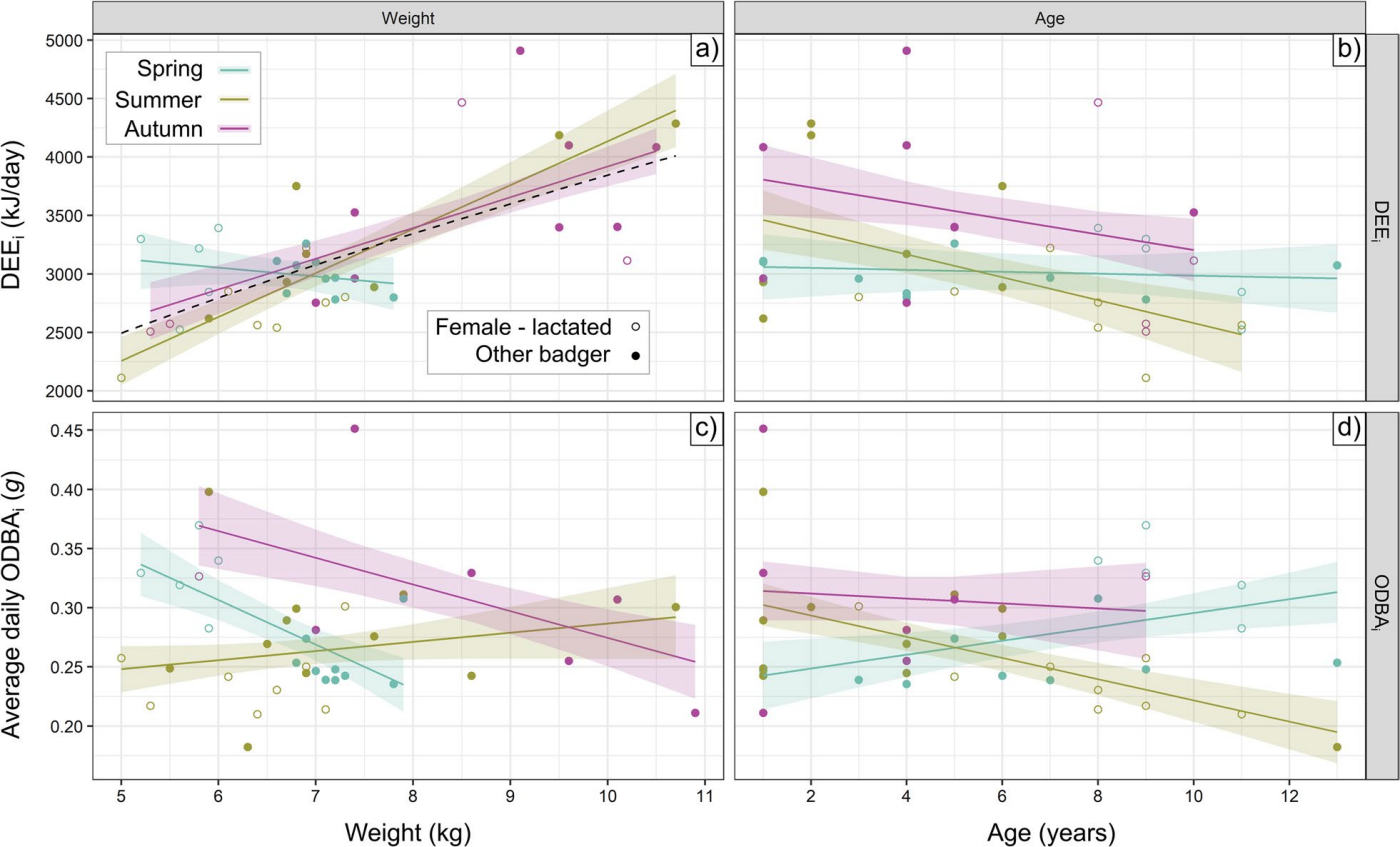
Intrinsic drivers of energy expenditure. Relationships between body weight **a**, **c** or age **b**, **d** and the two energy metrics (total energy expenditure, **a**-**b**, and mechanical energy expenditure, **c**-**d**) are shown (± SE). Dashed line in panel **a** depicts overall allometric scaling relationship, DEE_i_ α W^0.62^; open circles represent females that had lactated during the year studied, while closed circles represent all other badgers

To relax the assumption that each metric necessarily exhibited a positive power relationship, we further permitted the intercept to vary by season independent of this scaling equation. Recent research has noted the importance of testing exponential scaling relationships between DEE and body mass [55]. However, as the range of body weights spanned within this population was relatively small on a log-scale (0.76) and the fitted exponential curve strongly approximated a linear one (see Sect. "Drivers of ODBA and DEE", Fig. 1), all further modelling used the more parsimonious linear scale.

We performed linear regressions to model the response of DEE_i_ and ODBA_i_ separately as a function of body weight (to account for allometric effects as well as explanatory differences in capacity to engage in activity as a consequence of somatic energy stores, [4]), age, season (as a three-level unordered factor), and sex, as well as body weight:season and age:season interactions. As there were slight collinearities between body weight and age within season, we did not include these two terms in the same models. This resulted in four full models (two for DEE_i_ and two for ODBA_i_, including body weight and age in separate full-set models), subject to stepwise selection.

### Covariation of ODBA and DEE

Due to non-overlapping data recovery issues between energetic measurement methods (see Sect. "Energetic metric computation"), valid ODBA_i_ and DEE_i_ data coincided for 30 data records across 19 badgers (n spring = 12, summer = 13, autumn = 5; see Table 1). For these, we examined the relationship between the two metrics using linear models, at both the whole-study and seasonal scale. We controlled for the effects of weight on DEE_i_. We also calculated the seasonal ratio of total energy expenditure for each individual badger expressed as activity (OD_i_, where *i* represents an individual with overlapping ODBA_i_ and DEE_i_ observations, from *i* = 1 to *i* = 30) as the ratio ODBA_i_/DEE_i_.

**Table 1:** Data collection results

		Spring			Summer			Autumn		
		Deployed/	Recovered/	Retained	Deployed/	Recovered/	Retained	Recovered/	Deployed/	Retained
		Dosed	Recaptured		Dosed	Recaptured		Dosed	Recaptured	
Collars (ODBA)	2018	9	7	5	10	10	9	12	11	0
	2019	12	12	10	12	12	10	11	11	7
	sum	21	19	**15**	22	22	**19**	23	22	**7**
DLW	2018	9	6	5	11	11	8	12	8	5
	2019	12	11	9	12	10	6	11	11	8
	sum	21	17	**14**	23	21	**14**	23	19	**13**
Both	2018			4			7			0
	2019			8			6			5
	sum			**12**			**13**			**5**

Number of collars deployed with tri-axial accelerometers on badgers, number of badgers dosed with doubly-labelled water (DLW), and number of overlapping records for both methods in each season over two years. Successive columns show the initial sample size, the number of collars recovered or the number of badgers recaptured during valid DLW windows, and the number of retained data points after inspecting data quality. Total final seasonal sample sizes are bolded

### Energetic correlates of next-season body condition

Given our premise that reduced BCI represents the outcome of expending more energy than an individual consumes (i.e., a negative energy budget that will deplete their residual body fat stores), we investigated the relationship between seasonal DEE_i_, ODBA_i_ and OD_i_ and BCI in the season following energy measurement. Where possible, badgers were re-captured the following season, allowing this analysis for 25 out of 41 of ODBA_i_ records, 26 out of 41 of DEE_i_ records, and 19 out of 30 of the overlapping records. First, we calculated the residual (denoted by the subscript *res*) of each individual’s BCI in the season after energy assays (“next-season BCI_res_”) by controlling for the effects of age, calendar date, and sex in a generalized additive model (GAM, package *mgcv*, [56]). This GAM was constructed using BCI data from 27 years of badger captures from this population (n = 5866; see [4, 40]). Second, we constructed three linear models with ODBA_i_, DEE_i_, and OD_i_ as the predictor of next-season BCI_res_. Finally, we used BCI_res_-survival relationships modelled previously by Bright Ross et al. [4] to quantify the survival probability cost associated with any observed variation in individual BCI_res_, while holding population density and a series of weather conditions at the average levels for the studied site during the years with which the model was parameterised (1990–2016).

## Results

### Energetic metric computation

We recovered final blood samples within the 6–10 day anticipated valid recapture window for 57 of the 65 DLW dosings (n spring = 17, summer = 21, autumn = 19; Table 1), representing 29 unique badgers across two years. Of these, 2 samples had isotope enrichment at recovery too close to background to calculate DEE_i_ and a further 14 exhibited discrepancies consistent with improper dosing (likely related to the difficulties of injecting conscious animals), resulting in reliable DEE_i_ estimates for 41 dosings (representing 22 unique individuals with some repeated measures per individual across seasons; n spring = 14, summer = 14, autumn = 13; Table 1). We recovered 63 accelerometry collars, which due to a mix of hardware and software failures under field conditions produced 41 whole-period ODBA_i_ estimates (n spring = 15, summer = 19, autumn = 7; Table 1), with an average retained ODBA_i_ window of 5.3 days. ODBA_i_ and DEE_i_ values overlapped for 30 records (Table 1).

### Drivers of ODBA and DEE

*Season:* While mean ODBA_i_ did not differ substantially from season to season, it varied extensively between individuals in all seasons (standard deviation was 15.9%, 18.4%, and 24.4% of the mean in spring, summer, and autumn, and the most active badger had 1.6, 2.2, and 2.1 times higher ODBA_i_ than the least active badger in each season). DEE_i_ did not differ significantly between spring and summer (mean ± SD: spring = 3013 ± 240 kJ/day; summer = 3049 ± 630 kJ/day) but was significantly higher in autumn (3483 ± 767 kJ/day, difference from spring *p* = 0.046). There was substantial intra-season DEE_i_ variation between individuals, although intra-season variance was significantly lower (F-test for unequal variances) in spring (max difference of 1.3-fold in DEE_i_) than summer (2.0-fold max difference, F = 0.15, *p* = 0.001) or autumn (2.0-fold max difference, F = 0.10, *p* < 0.001). No significant overall difference was detected in either ODBA_i_ or DEE_i_ between 2018 and 2019 (*p*_*ODBA*_ = 0.86; *p*_*DEE*_ = 0.10).

*Scale allometry:* DEE_i_ was significantly and positively associated with body weight in summer and autumn (both *p* < 0.001), but not spring (*p* = 0.64; Fig. 1a). When modelled exponentially, DEE_i_ scaled allometrically with body weight to the -0.14-power in spring (not significantly different from no effect, *p* = 0.64), 0.90-power in summer (*p* < 0.001) and 0.65-power in autumn (*p* < 0.001). Pooling all seasons, DEE_i_ scaled to the 0.62 power (*p* < 0.001, R^2^ = 0.48, Fig. 1a). The different relationship during the spring was likely related to differential energy expenditure between females that had lactated that year (5/14 records were for reproductive females, which had relatively high DEE_i_ and low body weight, with a mean spring deviation of 350 kJ/day from the global allometric relationship) and other individuals (mean deviation = − 98 kJ/day). Conversely, there was a significant negative relationship between ODBA_i_ and body weight in spring (*p* = 0.02) and autumn (*p* = 0.04), with heavier badgers expending less mechanical energy (Fig. 1c); but not in summer (*p* = 0.38). The spring ODBA_i_-weight relationship was again in part related to reproductive status; females that had reproduced that spring (2/14 records were for reproductive females) had relatively high ODBA_i_ values (on average 1.3-fold higher than other badgers) and low body weight (Fig. 1c).

*Age:* DEE_i_ was lower for older badgers in summer (e.g., 9-year-olds expended 627 kJ/day less than 2-year-olds, on average, Fig. 1b), although this effect was only marginally significant (*p* = 0.054). In summer, older badgers were significantly (*p* < 0.001) less active than younger badgers (e.g., 1.4-fold higher ODBA_i_ in 2-year-olds than in 9-year-olds, Fig. 1d).

*Sex:* Sex was not retained in any model selection.

### Covariation of DEE and ODBA

**Fig. 2:**
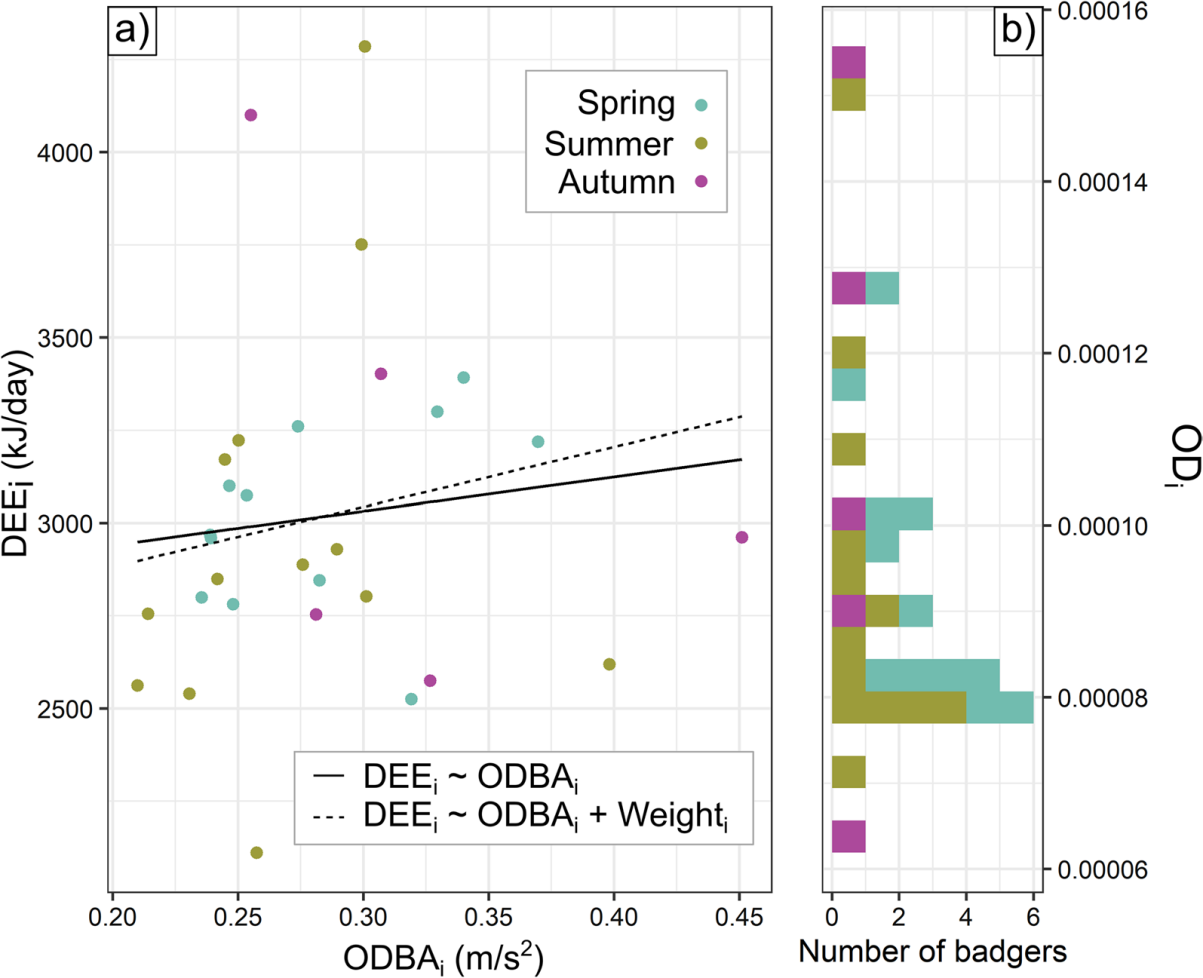
Covariation of DEE_i_ and ODBA_i_. Individuals **a** did not show a strong relationship between ODBA_i_ and DEE_i_ and **b** consequently exhibited widely varying ratios of ODBA_i_ to DEE_i_

We did not detect any overall relationship between average daily ODBA (ODBA_i_) and DEE_i_ (*p* = 0.56) or in any seasonal subset of these data, even after accounting for weight (*p* = 0.18, Fig. 2). However, the ratio of ODBA_i_ to DEE_i_ (OD_i_) varied substantially between individuals, with the highest seasonal values for individuals allocating 1.6x (spring), 2.2x (summer), or 2.4x (autumn) as much of their total energy to activity as the lowest ones (Fig. 2). In spring, 71% of variance in OD_i_ was explained by whether the badger was a female that had lactated: post-lactation females (5/12 records) expended 30% more of their total energy budget on ODBA_i_ than other badgers (Student’s *t*-test, *p* < 0.001).

### Drivers of next-season BCI

**Fig. 3:**
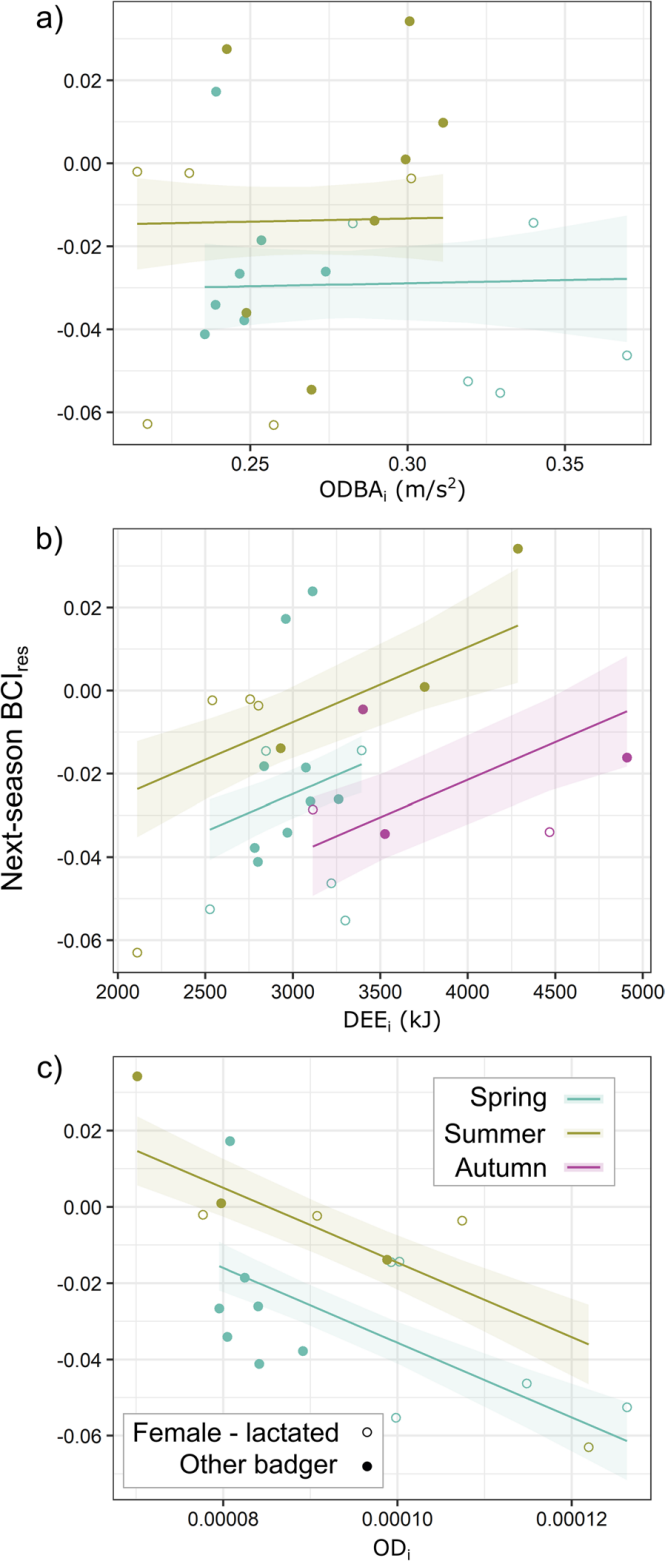
Energetic drivers of next-season BCI_res_. Lines show seasonal relationships (± SE) between next-season BCI_res_ and **a** average ODBA_i_, **b** DEE_i_, and **c** the ratio of ODBA_i_ to DEE_i_, while points show individual values. Open circles represent female badgers that had lactated during the year observed. BCI_res_ values are residuals from a GAM model (based on long-term population data) of BCI_i_ as a function of sex, age, and calendar date

Due to all collars failing during the first autumn’s assays, effects of autumn ODBA_i_ and OD_i_ on next-season BCI_res_ could not be tested. However, spring and summer ODBA_i_ exhibited no relationship with next-season BCI_res_ (*p* = 0.20, Fig. 3a). DEE_i_ showed a positive (Fig. 3b) but weakly significant correlation (*p* = 0.0496). However, spring and summer OD_i_ exhibited a strongly significant negative correlation with next-season BCI_res_ (Fig. 3c  *p* =  0.002, R^2^ = 0.50). While there was a positive association between an individual’s BCI_res_ in one season and the next, this association was not significant (*p* = 0.10).

**Fig. 4:**
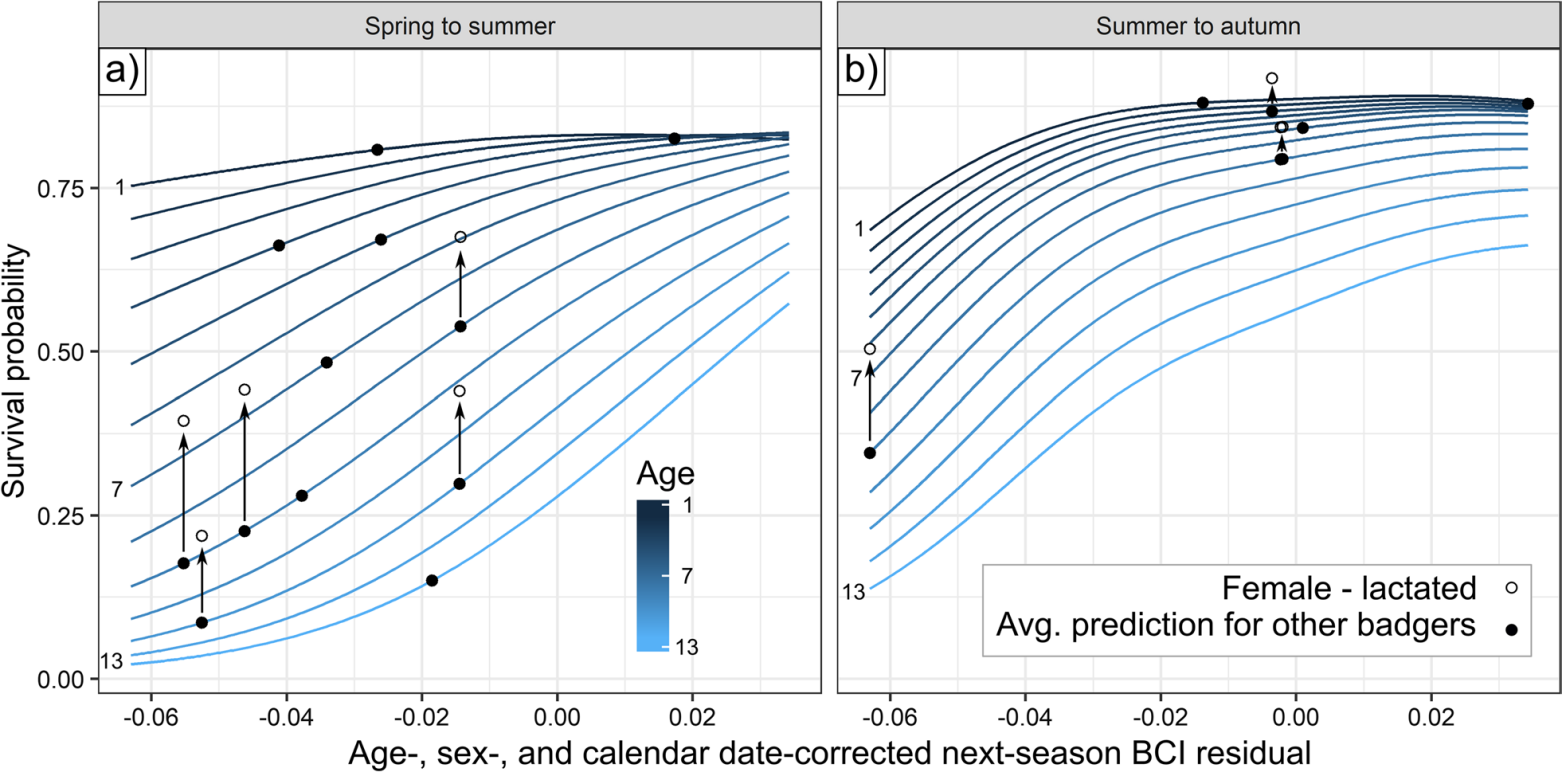
Survival costs from OD_i_-driven range of BCI_res_. Each line plots the average relationship for a badger of a given age between BCI_res_ in **a** summer and **b** autumn and that individual’s probability of surviving to the next year. Points show individual BCI_res_ values for the badgers in this study; individuals are placed on the line corresponding to their age at the time of the study. Post-reproductive females have weaker associations between BCI_res_ and survival probability [4]; therefore, they are shown as open circles, with the difference in survival probability due to their reproductive status represented by an arrow from a closed circle on their corresponding age curve

When examined in the context of population-level effects of BCI_res_ on survival probability, the range of next-season BCI_res_ values predicted by OD_i_ were associated with substantial differences in survival probability, depending on age—where survival probability is much more dependent on BCI_res_ for old badgers than for young badgers (Fig. 4). In a year with average population density and weather, the highest BCI_res_ predicted by the range of OD_i_ values we observed would have a 9.6% (in summer, prediction standard error -0.4–20.4%) or 23.1% (autumn, SE 13.6–33.1%) higher survival probability than the lowest BCI_res_ predicted for a 2-year-old; for a 9-year-old the same comparison spanned a much higher change in survival probability (summer: 29.8%, SE 17.5–40.5%; autumn: 44.9%, SE 31.0–57.2%). Figure 4 shows this relationship for all ages, with actual next-season BCI_res_ values (where we used the OD_i_-BCI_res_ trendline to generate the percentages in this section in order not to bias the results by projecting the survival of outliers) shown on the badger’s corresponding survival-age curve. Furthermore, reproducing (i.e., that had lactated that year, n = 5/12 in spring; 3/5 in summer) females exhibit higher survival probabilities for a given BCI_res_ than other badgers [4]; Fig. 4 highlights how much higher the survival probability is for the post-reproductive females in our dataset than it would be if they were a non-reproductive badger of the same age and BCI_res_.

## Discussion

We found substantial variability in both mechanical (ODBA_i_) and total (DEE_i_) energy expenditure among individual badgers experiencing the same environment simultaneously (Fig. 2), relating to different intrinsic traits. Heavier badgers expended up to ~ 1,500 kJ/day more than lighter badgers in summer and autumn. Young badgers exhibited up to 1.4x higher mechanical energy expenditure than old badgers in summer. Females that had lactated in early spring exhibited high DEE_i_ and ODBA_i_, driving a negative correlation between spring body weight and ODBA_i_ and cancelling out allometric scaling for spring DEE_i_ (Fig. 1). Crucially, we found a link between individual energy budgeting and lower next-season body condition (BCI_res_), whereby OD_i_ was more strongly correlated with BCI_res_ than either the active (ODBA_i_) or total (DEE_i_) energy budget of a given individual (Fig. 3). Interestingly, however, while the lower relative BCI associated with high OD_i_ values typically represent a substantially lower survival probability (Fig. 4), those badgers engaging in tactics reflective of high OD_i_ values were predominantly those whose life-history (being younger or being a lactating female) put them at less risk of mortality as a result. These insights were enabled by the rare intersection of insights from a long-term biometric dataset with activity-based and whole-organism energy measurements, each of which present difficulties in the wild (for which our attrition rate detailed in Sect. "Energetic metric computation" was in fact quite a success).

Wild populations are not monoclonal, but include shifting ratios of individuals with different life-histories [57] that experience different contemporary energetic contexts [2]. These differences lead to multiple optimal energy expenditure tactics among individuals experiencing the same prevailing conditions, due to individual-specific motivations (such as breeding) and sensitivities to stressors [58]. For instance, in all populations, some individuals are older than others. Age causes particular thermoregulatory challenges that can affect energy budgeting, with diminished thermogenic capacity [59], reduced capacity to dissipate heat [60], and diminished physiological capacity to cope with hyperthermia [61]. In addition, sarcopaenia [62] reduces the muscular efficiency of activity in older individuals; that is, an identical task takes more overall energy in the elderly. The impact of diminished efficiency should not be underestimated; as an illustrative example, one badger had to be excluded from our dataset because it developed a limp and consequently was only able to expend 73% of the average population ODBA_i_. Differences in the efficiency of activity can thus drive substantial differences in fitness in wild populations [18, 63].

Dens provide thermal refugia for fossorial animals [64], making non-emergence a behavioural alternative to autonomic thermoregulation [47, 65, 66]. Accordingly, one plausible explanation for reduced summer ODBA_i_ and (to a lesser extent) DEE_i_ in older badgers may be that they reduce non-essential activity during the summer and remain longer underground in their sett, given substantial variation in sett use by badgers [25, 67]. If elderly badgers were more active during the summer, higher ambient temperatures and lower water availability would cause them disproportionately greater energy costs than for younger badgers due to their geriatric thermoregulatory physiology [68]. This explanation is consistent with the combination of lower average body condition observed among older badgers [40] and the established greater cost to survival for older badgers from low body condition during the summer [4]. Minimizing unnecessary energy expenditure is important for these geriatric badgers: in older badgers, the loss of BCI associated with a higher fraction of total energy expenditure being spent on activity (evident as higher ODBA_i_) would typically be associated with up to a 44.9% reduction in survival probability according to the BCI_res_ model (Fig. 4).

Those badgers exhibiting high and costly OD_i_ ratios were predominantly lactating females. Female badgers that had reproduced in the year of study expended 350 kJ/day above allometric predictions in spring, even after lactation had ceased (Fig. 1). These same females were also more likely to have relatively high OD_i_ ratios and lower-than-average BCIs in subsequent seasons. Pertinently, however, our previous research has demonstrated that low body condition is less likely to result in mortality for reproductive females than for non-reproductive ones within this population (Fig. 4; [4]). While reproduction is an obligate component of fitness, in many longer-lived iteroparous species this substantial investment can be aborted and postponed until fairer conditions occur (where most reproductive-age females mate each year but far fewer produce cubs, [69]). Nevertheless, frailty to stressors increases with age and the trade-off between energy expended on current reproduction and postponed reproduction changes as individuals approach senescence [70], in some circumstances favouring a “terminal investment” [71].

In this same badger population, Sugianto et al. [72] found a decreasing female per capita reproductive rate after age 3. This coincided with the onset of somatic decline but came earlier than hormonal decline (5.5 years onwards—an age attained by 31.8% of females, or 55.8% of those attaining sexual maturity). This gradual decline eventually results in a return to pre-pubescent oestrone levels in the majority of females (functionally reaching menopause) around age 9, with an average post-reproductive lifespan of 2.6 years [72], evidencing the difficulty of meeting both the endocrinological and somatic requirements of reproduction at advanced age [73]. Nevertheless, very old female badgers (age 9 +) exhibit heterogeneous endocrinological profiles [72], with some seemingly escaping the reproductive constraints of age; parallel research has also found that comparatively “higher-quality” reproductive older female badgers have higher survival probability than other females of an equivalent age [4]. Congruent with this, we detected moderately more variation between individuals spring ODBA_i_ (this being the season most closely aligned with mating) in badgers aged 8 and older than in younger badgers (Fig. 1d), suggesting some old females—likely those still capable of reproduction—engaged in energy budgets that involved more activity than others.

We found a direct cost to body condition associated with elevated OD_i_ ratios (Fig. 3c). Expending energy on voluntary activity redirects resources from growth and somatic maintenance [1], but also tends to increase basal metabolic rate [74]. More costly energy budgets typically incur life-history costs, where higher total energy expenditure correlates with a shorter realised lifespan within taxa [75, 76]. Parsing causality in somatic-energetic correlative observations (Fig. 3) presents a challenge: do heavier, fatter badgers reduce activity because they do not need to forage as much, thus remaining heavier; or do heavier badgers maintain somatic stores because they do not engage in an excess of voluntary activity? Both explanations are plausible, although it is notable in this context that white adipose tissue is relatively metabolically inactive [77]. Heavier badgers expend up to twice as much total energy as light ones, despite no concurrent activity elevation (Fig. 1), due to allometric scaling effects [78]. Conversely, a substantial portion of lighter badgers (with lower DEE_i_) spent a higher fraction of their total energy expenditure on activity (Figs. 1, 3)—including all reproductive females during the spring. While the drawbacks of excess fat have been examined in the context of agility [39, 79], the elevated maintenance costs of excess somatic stores (including both adipose tissue and greater muscular bulk) have received little attention in species for which energy stores are linked to survival at a coarse scale (compared with, for instance, small passerines, [80]). This is crucial because if the added bulk does not contribute to reproductive output, additional maintenance costs over longer durations may not enhance that individual’s lifetime fitness [81].

According to the Peak Demands model of energy budgeting, animals can elevate their total energy expenditure to take advantage of either high and predictable food availability or minimal seasonal energy losses—this elevated energy expenditure may be activity-based (e.g., enabling additional foraging, [82], or mate-searching, [83]) and/or physiological (e.g. aligning lactation with peak food availability, [22]). In contrast, according to the Reallocation model, animals may rebalance allocations to maintain a steady expenditure throughout time, particularly when energy availability/losses do not vary predictably [84, 85]. While these models can be evaluated across species, it is pertinent to consider how contextual energetic fragility might affect an individual’s ability to conform to a seasonal spike in energy expenditure (Peak Demands model) or maintain an equivalent allocation over time (Reallocation model). For instance, the first free-ranging DEE study on badgers found support for the Reallocation model, with no significant seasonal differences in energy expenditure [53]. However, in a species that experiences high seasonal spikes in energy demand, it may instead be that averages are comprised of individuals following combinations of Peak Demands or Reallocation models, depending on their individual energetic context. Several studies in other species have also found substantial variation from these models, both between individuals [22, 23] and over time [86].

Populations are comprised of individuals with different energy ceilings (summit metabolic throughput, [24]) and floors (basal requirements, [87]), dictating diverse energy-budgeting tactics. As energy budgets and related metrics determine the efficiency and fitness benefits of different activities (e.g., [21, 88]), metabolic traits co-vary within populations with a suite of behavioural types (according to the extended pace-of-life syndrome hypothesis, [20, 89]). Nevertheless, while basal, resting, and even maximum metabolic rates are heritable [90, 91], DEE is not [92]. As environmental conditions vary and drive different availabilities of and obligate needs for energy, the optimal basal metabolic rate varies as well [93]. Therefore, while individuals may be consistently constrained by their underlying basal metabolism, its interactions with the environment may produce very different energetic outcomes. In badgers, this is evident in BCI, which is not highly repeatable for most individuals [4]. Therefore, while the lack of a clear survival cost to reproduction for the low-BCI females in our study could be attributed to individual quality [94, 95], it more likely reflects the co-existence of individuals with different energetic contexts within the population at the same time, pursuing high-OD tactics when they are able and scaling back unnecessary activity when they must.

The presence of these different tactics within a single population is relevant to the long-term resilience of that population. Under the more frequent and severe disturbances associated with human-induced rapid environmental change (HIREC, [96]), fewer animals have energetic surpluses to pursue the risky energy-partitioning tactics associated with high OD ratios. Under these stressful conditions, energy efficiency is prioritised [63] and stability dominates selection (although populations may converge on diverse solutions to energy efficiency, [97]). It is well-understood that phenotypic diversity is beneficial to the resilience of populations and communities under strain (the “portfolio effect”, [98, 99]). Less attention has been paid to how populations respond energetically to severe and persistent environmental stressors. Nevertheless, many species have survived rapid, sustained changes in environmental conditions in their palaeontological record without extinction (e.g. Dansgaard-Oeschger events, [100]), and exhibit the hallmarks of that resilience today in both the diversity of internal state within populations (e.g., hormonal titres and associated energy expenditure, [101]) and the plasticity of individual behavioural responses to energy availability [102]. For the conservation of present and future biodiversity, characterising the diversity of inter-individual energetic tactics within wild populations, as well as the generators of that diversity, will enable a better mechanistic understanding of population resilience to HIREC, and inform better targeted management interventions.

## Conclusions

Within the same population of free-living badgers and under the same environmental conditions, we found wide variation in both mechanical and total energy expenditure. This variation correlated with both age and reproductive status, and affected next-season body condition. Despite our small sample size, we found that the riskiness of adopted energetic tactics depends on how influential the resulting next-season body-condition decreases were on survival probability. These findings contribute to a growing understanding that populations can pivot under new environmental conditions due to the mix of facultative and fixed energetic tactics undertaken by those individuals comprising the population. It appears that wild populations will attempt to utilize inter-individual heterogeneity in energetic tactics to adapt, whether to a greater or lesser extent, to human-induced rapid environmental change; further research into the evolutionary dynamics of this energetic heterogeneity will be key to elucidating how successful that adaptation will be.

## Data Availability

The data used in analyses for this study will be stripped of unique identifying information and made available at the Wytham Woods Badger Project dataverse (https://dataverse.harvard.edu/dataverse/wytham-badgers) upon acceptance for publication.

## Abbreviations

BCI: Body-condition index.
DEE: Daily energy expenditure.
DIE: Desirable initial enrichment.
DLW: Doubly-labelled water.
GAM: Generalised additive model.
HIREC: Human-induced rapid environmental change.
IE: Injectate enrichment.
ODBA: Overall dynamic body acceleration.
OD: The ratio of overall dynamic body acceleration to daily energy expenditure
